# Site-Specific Glycan-Masking/Unmasking Hemagglutinin Antigen Design to Elicit Broadly Neutralizing and Stem-Binding Antibodies Against Highly Pathogenic Avian Influenza H5N1 Virus Infections

**DOI:** 10.3389/fimmu.2021.692700

**Published:** 2021-07-14

**Authors:** Ting-Hsuan Chen, Ya-Lin Yang, Jia-Tsrong Jan, Chung-Chu Chen, Suh-Chin Wu

**Affiliations:** ^1^ Institute of Biotechnology, National Tsing Hua University, Hsinchu, Taiwan; ^2^ Genomics Research Center, Academia Sinica, Taipei, Taiwan; ^3^ Department of Internal Medicine, MacKay Memorial Hospital, Hsinchu, Taiwan; ^4^ Teaching Center of Natural Science, Minghsin University of Science and Technology, Hsinchu, Taiwan; ^5^ Department of Medical Science, National Tsing Hua University, Hsinchu, Taiwan

**Keywords:** hemagglutinin, glycan masking, glycan unmasking, H5N1, vaccine

## Abstract

The highly pathogenic avian influenza (HPAI) H5N1 viruses with the capability of transmission from birds to humans have a serious impact on public health. To date, HPAI H5N1 viruses have evolved into ten antigenically distinct clades that could cause a mismatch of vaccine strains and reduce vaccine efficacy. In this study, the glycan masking and unmasking strategies on hemagglutinin antigen were used for designing two antigens: H5-dm/st2 and H5-tm/st2, and investigated for their elicited immunity using two-dose recombinant H5 (rH5) immunization and a first-dose adenovirus vector prime, followed by a second-dose rH5 protein booster immunization. The H5-dm/st2 antigen was found to elicit broadly neutralizing antibodies against different H5N1 clade/subclade viruses, as well as more stem-binding antibodies to inhibit HA-facilitated membrane fusion activity. Mice immunized with the H5-dm/st2 antigen had a higher survival rate when challenged with homologous and heterologous clades of H5N1 viruses. Mutant influenza virus replaced with the H5-dm/st2 gene generated by reverse genetics (RG) technology amplified well in MDCK cells and embryonated chicken eggs. Again, the inactivated H5N1-dm/st2 RG virus elicited more potent cross-clade neutralizing and anti-fusion antibodies in sera. Therefore, the H5N1-dm/st2 RG virus with the site-specific glycan-masking on the globular head and the glycan-unmasking on the stem region of H5 antigen can be used for further development of cross-protective H5N1 vaccines.

## Introduction

Highly pathogenic avian influenza (HPAI) H5N1 viruses, which are transmitted from birds to humans, have a serious impact on public health (1–3). After the first outbreak in Hong Kong in 1997, the HPAI H5N1 viruses re-emerged in 2003 and have continued to spread from Asia to Europe and Africa, with a total of 861 human infection cases and an approximately 53% mortality rate (4). Phylogenic analysis of H5 hemagglutinin (HA) revealed that HPAI H5N1 viruses have evolved into ten antigenically distinct clades that may cause antigenic mismatches for selecting the vaccine strain(s) (5, 6). No vaccine strains have been shown to elicit cross-protective immunity against a wide range of H5N1 clades and subclades (7, 8). The World Health Organization (WHO) has recommended 32 candidate vaccine viruses of H5N1 virus clades/subclades for vaccine preparation (9). Therefore, the development of broadly protective H5N1 vaccines is of particular interest to control these distinct antigenic H5N1 clade/subclade virus infections.

The influenza HA antigen is a major envelope glycoprotein accounting for approximately 80% of all spikes in influenza virions. It is considered as the major antigen content for characterizing influenza vaccines. The HA glycoprotein has a trimeric structure with several N-glycans, and each monomer consists of two parts: a globular head and a stem region that are folded within six disulfide bonds (10). The N-linked glycosylation sites on the globular head vary among different strains and subtypes (11). In contrast, the N-linked glycosylation sites in the stem region are mostly well-conserved among various influenza virus strains (12). Acquisition of additional N-glycan modifications particularly in the globular head has evolved as a strategy for seasonal H1N1 and H3N2 viruses to avoid human immune responses (13, 14). We previously reported that glycan-masking H5 antigens in the HA globular head region with (i) triple mutations (H5-tm) on residues 83 (83ANP85 replaced by 83NNT85), 127 (127ASL129 replaced by 127NSS129) and 138 (138QRK140 replaced by 138NGT140), or with (ii) double mutations (H5-dm) on residues 127 (127ASL129 replaced by 127NSS129) and 138 (138QRK140 replaced by 138NGT140) elicited broader neutralizing antibodies against heterologous clades/subclades of HPAI H5N1 viruses (15–17). We also demonstrated that glycan-unmasking H5 antigen with a single mutation in the HA stem region (HA-st2) on residue 484 (484NGT486 replaced by 484AGT486) elicited more potent neutralizing antibodies against homologous, heterologous and heterosubtypic viruses (18). The glycan unmasked H5 antigen (HA-st2) also provided a significantly improvement in the protection against homologous virus challenges, but to a less degree for the protection against heterosubtypic pH1N1 virus challenges (18). In the present study, we investigated the use of a combination of H5 globular head glycan-masking antigens and the H5 stem glycan-unmasking antigens: (i) H5-dm/st2 with 127NSS129, 138NGT140, and 484AGT486 mutations and (ii) H5-tm/st2 with 83NNT85, 127NSS129, 138NGT140, 484AGT486 mutations. Two types of immunization regimens were investigated in BALB/c mice, including two-dose recombinant H5 (rH5) immunization and a first-dose adenovirus vector prime, followed by a second-dose rH5 protein booster. Immune responses were measured for the elicitation of H5-specfiic IgG, virus-neutralizing antibodies, stem-binding antibodies, anti-fusion antibodies, and the protection against different clade/subclades of H5N1 viruses. We also obtained the reverse genetics (RG) viruses of H5N1-wt and H5N1-dm/st2 using a PR8-based 8 plasmid system. The growth kinetics in MDCK cells, virus yields in embryonated chicken eggs, and formalin inactivation kinetics were examined for H5N1-wt and H5N1-dm/st2 RG viruses. Immune responses elicited by the inactivated RG viruses were determined by measuring neutralizing and anti-fusion antibodies in sera. The results indicated that H5N1-dm/st2 RG virus with the glycan-masking on the HA globular head and the glycan-unmasking on the HA stem region can be used for further development of cross-clade H5N1 vaccines.

## Materials and Methods

### Expression and Purification of rH5 Proteins

Insect cell codon-optimized H5 cDNA sequence of the A/Thailand/1(KAN-1)/2004(H5N1) virus strain (GenBank: CY111598.1) was obtained from Genomics BioSci & Tech. Ltd., Taiwan to construct the soluble rH5 protein expression plasmids. The C-terminal cytoplasmic and transmembrane domains of full-length HA were replaced with a GCN4-pII leucine zipper (MKQIEDKIEEILSKIYHIENEIARIKKLIGEV) for trimerization, a thrombin cleavage site, and a His-tag for purification as previously described (18, 19). The rH5-dm/st2 gene was constructed by site-directed PCR to introduce additional N-linked glycosylation motifs (N-X-S/T) on residue 127 (127ASL replaced by 127NSS), and 138 (138QGK replaced by 138NGT), and a native N-linked glycan in the stem region was removed by mutation of 484NGT to 484AGT. rH5-tm/st2 was further constructed by adding an N-linked glycosylation motif on residue 83 (83ASL replaced by 83NSS). The Bac-to-Bac System (Invitrogen) was used to obtain recombinant baculoviruses carrying rH5-wt, rH5-dm/st2, and rH5-tm/st2 respectively according to the manufacturer’s instructions. For large-scale production, Sf9 cells were grown in 600 ml SF900-II serum-free medium (Invitrogen) at a concentration of 2×10^6^ cells/ml, and then infected with the respective recombinant baculovirus at 3 MOI. The culture supernatant was collected 72 h post-infection, and then rH5-wt, rH5-dm/st2, and rH5-tm/st2 proteins were purified using nickel-chelated affinity chromatography (Tosoh), dialyzed with PBS, and stored at -20°C. The purity and molecular weight were determined by SDS-PAGE and western blotting using anti-H5 antibody. The sialic acid-binding activity of each protein was tested by a typical hemagglutination assay using 0.5% turkey RBCs.

### H5-Specific Monoclonal Antibody Bindings by ELISA

Plates (96-well) were coated with 0.2 µg bovine serum albumin (BSA), or rH5-wt, rH5-dm/st2, or rH5-tm/str2 proteins, incubated overnight at 4°C, and blocked with 200 µl blocking buffer (PBS with 1% BSA) for 2 hours at room temperature. Two-fold serially diluted samples of monoclonal antibodies, including 9E8, F10, C179, CR6261, and FI6V3 were added to each well and incubated for 1 h. Horseradish peroxidase (HRP)-conjugated anti-mouse or anti-human IgG antibodies (GeneTex) and TMB substrate (BioLegend) were used to develop signals, reactions were stopped using 2N H_2_SO_4_. The optical density at 450 nm was measured using a TECAN spectrophotometer. The end-point titration values were calculated by a final serial dilution higher than 0.2 in the optical density value.

### Fetuin Binding Assay

Individual wells in 96-well plates were coated with 50 μg/ml of fetuin (Sigma), held overnight at 4°C, and blocked with 1% BSA in PBS buffer followed by three washes with 0.05% Tween 20/PBS buffer. Serially diluted soluble rH5 proteins were pre-mixed with HRP-conjugated anti-His tag antibodies (Bethyl Laboratories, Inc.) for 30 min, added to individual plates, and incubated for 60 min at room temperature. After three additional washes, H5 binding was detected by ELISA (450 nm OD).

### Construction of Adenovirus Vectors

The ViraPower Adenoviral Expression System (Invitrogen) was used to create adenovirus vectors containing the full-length codon-optimized H5HA based on the A/Thailand/1(KAN-1)/2004 strain with a cleavage site mutation to retain un-cleaved proteins as previously reported (16). The H5-dm/st2 and H5-tm/st2 genes were obtained by site-directed PCR. The respective pENTR vectors containing the H5-wt, H5-dm/st2, and H5-tm/st2 genes were site-directly recombined with pAd/CMV/V5-DEST vectors by LR Clonase Enzyme Mix (Invitrogen) for generating the plasmids for transfection. After Pac I digestion, the recombined pAd/CMV/V5-DEST vectors were transfected into HEK293A cells (Invitrogen) for replication-incompetent adenovirus vector production. Recombinant adenoviruses vectors were collected 14 days post-transfection, and virus titers were determined by plaque assays. H5-wt, H5-dm/st2, and H5-tm/st2 protein expressions in cells infected with the respective recombinant adenoviruses were confirmed by SDS-PAGE and Western blotting using anti-H5 antibodies. Hemadsoption contributed by HA proteins expressed by adenovirus infected cells was confirmed by hemadsorption assay (15). 5x10^5^ HEK293A cells were infected with 10^6^ PFU for 48 h, washed with PBS and co-incubated with 0.5% turkey red blood cells (RBC) for 30 min. And then, the cells were washed with PBS, RBCs absorbed on the cell surface were observed by a microscopy (Olympus IX70, Olympus).

### Mouse Immunizations

Groups of female BALB/c mice (6–8 weeks old; 5 mice per group) were intramuscularly immunized by two different immunization regimens: (i) two-dose immunizations with 20 μg rH5 proteins formulated in PELC/CpG adjuvant or (ii) first-dose priming with 10^8^ pfu Ad-H5 followed by second-dose booster with 20 μg rH5 proteins plus PELC/CpG adjuvant, all in a three-week interval. We have previously reported the use of PELC, a squalene-based oil-in-water emulsion adjuvant system, combined with K3 CpG ODN for rH5 protein immunizations eliciting more potent neutralizing antibodies in mice (19). Antisera were collected two weeks after the second dose immunizations, incubated at 56°C for 30 min for complement inactivation, and stored at -20°C. All procedures involving animals were performed in accordance with the guidelines established by the Laboratory Animal Center of National Tsing Hua University (NTHU). Animal use protocols were reviewed and approved by the NTHU Institutional Animal Care and Use Committee (approval no. 108044).

### H5-Specific Antisera Titer by ELISA

Plates (96-well) were coated with 0.2 µg rH5-wt protein or 0.2 µg H5N1 (RG-14) inactivated virus, incubated overnight at 4°C, and blocked with 200 µl blocking buffer (PBS with 1% BSA) for 2 hours at room temperature. Two-fold serially serum samples were added to each well and incubated for 1 h. Horseradish peroxidase (HRP) conjugated anti-mouse IgG antibody (GeneTex) and TMB substrate (BioLegend) were used to develop signals, followed by stopping the reaction by 2N H_2_SO_4_. Optical density at 450 nm was measured using a TECAN spectrophotometer.

### H5pp Construction for Neutralization Assay

For generating H5pp particles, three plasmids including (i) pcDNA3.1(+) plasmids expressing H5HA of A/Thailand/1(KAN-1)/2004(H5N1) (clade 1), A/bar-headed goose/Qinghai/1A/2005(H5N1) (clade 2.2), A/Hubei/1/2010 (H5N1) (clade 2.3.2.1a), or A/Anhui/1/2005(H5N1) (clade 2.3.4), (ii) pcDNA3.1(+) plasmid expressing N1NA of A/Thailand/1(KAN-1)/2004(H5N1), and (iii) pNL Luc E^-^ R^-^ plasmid were co-transfected to 293T cells as described previously (16, 20). 50 μl of two-fold serially diluted serum samples (starting at 1:10) were incubated with 1 TCID50 of H5pp at 37°C for 1 h, and then the mixtures were incubated with 1.5×10^5^ MDCK cells per well at 37°C for 48 h. The H5pp-infected cells were lysed using Glo lysis buffer (Promega), and treated with neolite luciferase substrate (PerkinElmer) for luciferase activity measurements. The relative light units (RLU) values developed from the group without serum and the group without H5pp were defined as 0% and 100% neutralization respectively. The RLU values of the other groups were normalized for neutralization percentage calculation.

### Protein Absorption and Monoclonal Antibody Competition Assays

To measure the stem‐specific antibody titers in serum samples, a mutant rH5 protein (Δstem‐rH5) containing an additional N‐linked glycosylation site (^375^IDG replaced by ^375^NDT) in the mid‐stem helix A region was constructed and produced by Sf9 cells as previously reported (16, 18, 21). The Δstem‐rH5 protein was used to remove non-stem-binding antibodies in the sera. For the protein absorption assay, Δstem‐rH5 protein (40 μg/ml) coupled Ni‐nitrilotriacetic acid (NTA) beads were incubated with serum samples overnight at 4°C in 1.5 ml tubes for non-stem‐specific antibody absorption. After centrifugation, the supernatants of preabsorbed serum samples were transferred to ELISA plates coated with rH5-wt proteins of the KAN-1, Qinghai, Hubei, and Anhui strains. Stem‐specific IgG titers were measured by ELISA. For the CR6261 and FI6v3 stem-binding mAb competition assay, the Δstem‐rH5-preabsorbed sera were first mixed with 2-fold-diluted monoclonal antibodies CR6261 or FI6v3 (starting from 10 μg/ml) and then incubated in ELISA plates precoated with rH5 proteins. After 1 h of incubation, the IgG titers were measured by ELISA for competition level calculations.

### Membrane Fusion Inhibition Assay

Membrane fusion inhibition activity against H5N1 virus was determined by luciferase-based cell-cell fusion assays as previously described (18, 22, 23). In brief, donor 293T cells (2 ×10^4^ cells per well in 96-well plates) were transfected with pcDNA3.1 plasmid containing the H5 gene of A/Thailand/1(KAN-1)/2004(H5N1) and pCAGT7pol plasmid. Indicator 293 T cells (9 ×10^5^ cells per well in 6-well plates) were transfected with phRL-TK (Promega) and pT7EMCVLuc plasmids which contains a firefly luciferase and a sea pansy Renilla luciferase coding sequence respectively. Renilla luciferase activity was used to confirm transfection efficiency. At 48 h post-transfection, indicator cells were detached from the plate and added to the donor cells and incubated with diluted serum samples (in MEM-α containing 1.0 μg/ml trypsin-TPCK). After 1 h of incubation at 37°C, the cells were washed and incubated with low-pH fusion buffer (PBS, pH 4.9). After 5 min of incubation at 37°C, the supernatant was replaced with the standard growth medium. After another 7 h of incubation at 37°C, a dual-luciferase reporter assay system (Promega) was used to develop the signals. The relative luminescence unit (RLU) was measured using a Victor 3 plate reader (PerkinElmer). The signal of the negative control (transfected indicator cells alone) was defined as 1, and sample signals were calculated as normalized firefly luciferase value/normalized Renilla luciferase value.

### Viral Challenges

The immunized mice were anesthetized and intranasally challenged with 10-fold of the MLD50 of the reassortant H5N1 virus of NIBRG‐14 [clade 1, A/Vietnam/1194/2004 (RG‐14)], IBCDC-RG-2 [clade 2.3.1, A/Indonesia/5/05-like (RG-2)], or NIBRG‐23 [A/turkey/Turkey/1/05; clade 2.2 (RG‐23)] three weeks after the final immunization. Groups of PBS‐immunized mice were used as the negative control. The body weight and survival rate were recorded for 14 days. The protocols were reviewed and approved by the IACUC of Academia Sinica, Taiwan. According to the IACUC guidelines, a body weight < 75% was recognized as the endpoint. The challenge experiments were performed in a biosafety level 2+ (BSL‐2+) enhancement facility.

### Generation of RG Viruses

The sequences of PB, PB1, PA, NP, M, and NS from A/PR/8/1934(H1N1) and those of HA and NA from A/Viet Nam/1203/2004(H5N1) were cloned into modified pcDNA3.1 plasmid containing an RNA polymerase II promoter (CMV promoter) and a human RNA polymerase I promoter (PolIp) similar to the generation of pHW2000 (24). The poly basic cleavage site of HA was replaced with a threonine to meet biosafety requirements (25). Plasmids containing mutant H5 genes (H5-dm/st2 and H5-tm/st2) were obtained by site-directed PCR. The eight pFlu plasmids (1 µg each) were co-transfected into a MDCK/293T (4.0-4.5 x 10^5^) co-culture in a 6 well plate using TransIT^®^-LT1 Transfection Reagent (Mirus Bio) according to the manufactures’ instructions. The medium was changed to OPTI-MEM with 0.5 µg/ml TPCK-trypsin 24 h after transfection. After an additional 72 h incubation at 37°C, the supernatant was collected and tested by hemagglutination assay to confirm virus rescue. The rescued viruses were further amplified in embryonated chicken eggs and MDCK cells. Virus titers were determined using plaque assay. To measure virus replication curves, 2x10^7^ MDCK cells were infected with each virus at MOI = 0.001 in MEM-α + 0.5 µg/ml TPCK-trypsin for 1 h, washed with PBS, and incubated in MEM-α + 0.5 µg/ml TPCK-trypsin. Virus titers of samples collected every 12 h were determined by plaque assays and plotted as replication kinetic curves.

### Plaque Assay

Serially diluted virus samples in MEM-α with 0.5 µg/ml TPCK-trypsin were added to 9.5 x10^5^ MDCK cells in 6-well plates prepared 48 h in advance. After 1 h incubation at 37^0^C for virus absorption, the supernatants were removed and the cells were washed with PBS. 3 ml of gel overlay (0.5% low melting agarose in MEM-α with 0.5 µg/ml TPCK-trypsin) was added to each well. After further incubation at 37°C for 48 h, the cells were fixed with 4% formalin (Sigma) for 6 h, and then plaques were stained with 1% crystal violet.

### Inactivated RG Virus Preparation and Immunization

After 72 h of incubation at 37°C, 200 ml of virus-containing supernatant was collected from virus-infected MDCK culture (MOI=0.001) and treated with 0.01% formalin (Sigma) at 4^0^C and shaken at 200 rpm for 24 h. During this inactivation period, virus samples were collected at 0, 2, 4, and 6 h post-formalin treatment to determine inactivation kinetic curves by plaque assays. HA titer of samples collected at 24 h post-inactivation was determined using RBC hemagglutination assay. The remaining inactivated viruses were concentrated to 30 ml using a 100 KDa spin column (Millipore), and were added on top of 5 ml of a 20% sucrose solution (wt/vol) prepared in TNE buffer (0.1M NaCl, 1mM EDTA, 10 mM Tris-HCl, pH=7.4) in six ultracentrifugation tubes (Hitachi). After ultracentrifugation at 82700 xg at 4°C for 2 h, pellets were dissolved in 600 ul PBS, aliquoted, and stored at -80°C (26, 27). The total protein concentrations of inactivated viruses were determined by the Bradford protein assay (Bio-rad). Groups of female BALB/c mice (6–8 weeks old; 5 mice per group) were intramuscularly immunized with two doses of inactivated viruses containing 0.2 µg HA formulated with 300 µg Al(OH)_3_ (Alhydrogel adjuvant, Invivogen) with a three week interval. Antisera were collected two weeks after the second dose immunization, incubated at 56°C for 30 min for complement inactivation, and stored at -20°C.

### Statistical Analyses

All results were analyzed using one‐way analysis of variance (ANOVA) using the GraphPad Prism v6.01. Statistical significance in all results is expressed as the following: *p < 0.05; **p < 0.01; ***p < 0.001; and ***p < 0.0001. All experiments were performed at least twice.

## Results

### Construction, Expression, and Characterization of Soluble rH5 Proteins With the Combination of Glycan-Masking and Glycan-Unmasking HA Antigen Design

**Figure 1:**
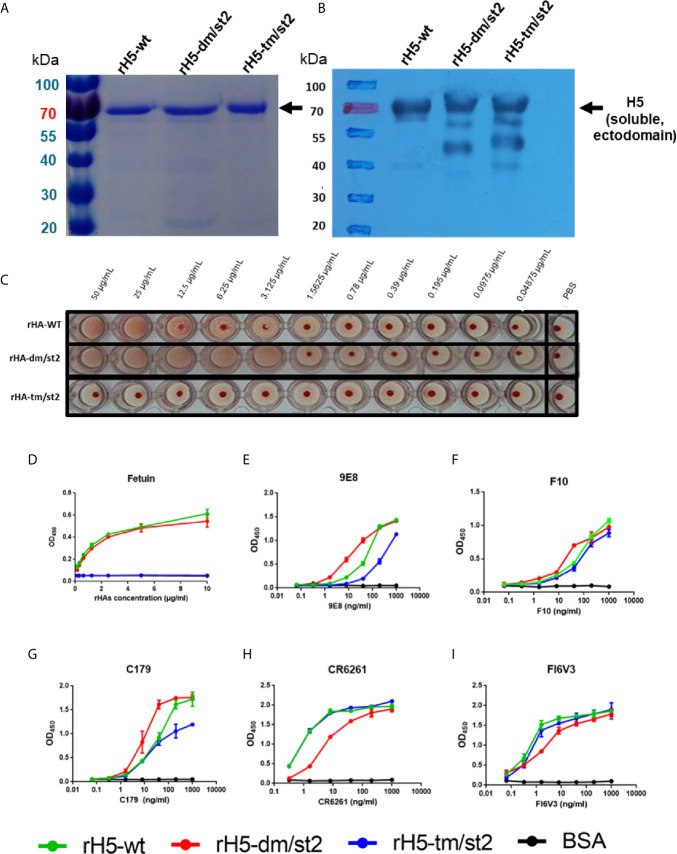
Characterizations of rH5-wt, rH5-dm/st2, rH5-tm/st2 proteins. The purified rH5-wt, rH5-dm/st2, and rH5-tm/st2 proteins were characterized by **(A)** SDS-PAGE and **(B)** western blotting. The sialic acid bindings of rH5 proteins were determined by **(C)** RBC hemagglutination and **(D)** fetuin binding assay. Bindings of rH5-wt, rH5-dm/st2, and rH5-tm/st2 proteins with **(E)** 9E8, **(F)** F10, **(G)** C179, **(H)** CR6261, and **(I)** FI6V3 mAbs were determined by ELISA.

To design a combination of the site-specific glycan-masking and glycan-unmasking antigens that we previously reported (15–18), we constructed rH5 proteins with the following mutations: (i) rH5-dm/st2 (127NSS129 + 138NGT140 + 484AGT486) and (ii) rH5-tm/st2 (83NSS85 + 127NSS129 + 138NGT140 + 484AGT486) (**Figure S1**). The wild-type (wt) and the two rH5 antigens (rH5-dm/st2 and rH5-tm/st2) were expressed in baculovirus-infected Sf9 insect cells and purified using nickel-chelated affinity chromatography. Purified proteins were analyzed on SDS-PAGE gels with Coomassie blue staining and western blotting, with the molecular weights of at approximately 70 kDa for rH5 and slightly higher than 70 kDa for rH5-dm/st2 and rH5-dm/st (**Figures 1A, B**). These purified H5 proteins were tested for red blood cell (RBC) hemagglutination, and the results indicated that rH5-dm/st2 had an approximately 3-log increase in RBC hemagglutination compared to that of rH5-wt and no hemagglutination for rH5-tm/st2 (**Figure 1C**). These results were confirmed by a fetuin-binding ELISA assay, showing similar dose-dependent binding curves between rH5-wt and rH5-dm/st2 in contrast to the absence of bindings for rH5-tm/st2 and the BSA control (**Figure 1D**). Several monoclonal antibodies (mAbs) targeting the globular head receptor binding site (mAb 9E8) or the stem region (mAbs F10, C179, CR6261, FI6V3) were used to measure their binding to rH5-wt, rH5-dm/st2, and rH5-tm/st2 (**Figures 1E–I**). The rH5-tm/st2 protein had relatively lower binding to the globular head-specific mAb 9E8 (190 loop of receptor binding site) compared to rH5-wt and rH5-dm/st2 (**Figure 1E**). The rH5-dm/st2 protein had slightly reduced binding to the two stem-binding mAb CR6261 and FI6V3 (**Figures 1H, I**). Overall, the rH5-dm/st2 and rH5-tm/st2 antigens retained their binding by these mAbs.

### Construction of Adenovirus Vectors Encoding the Full-Length H5 Gene With the Combination of Glycan-Masking and Glycan-Unmasking HA Antigen Design

**Figure 2:**
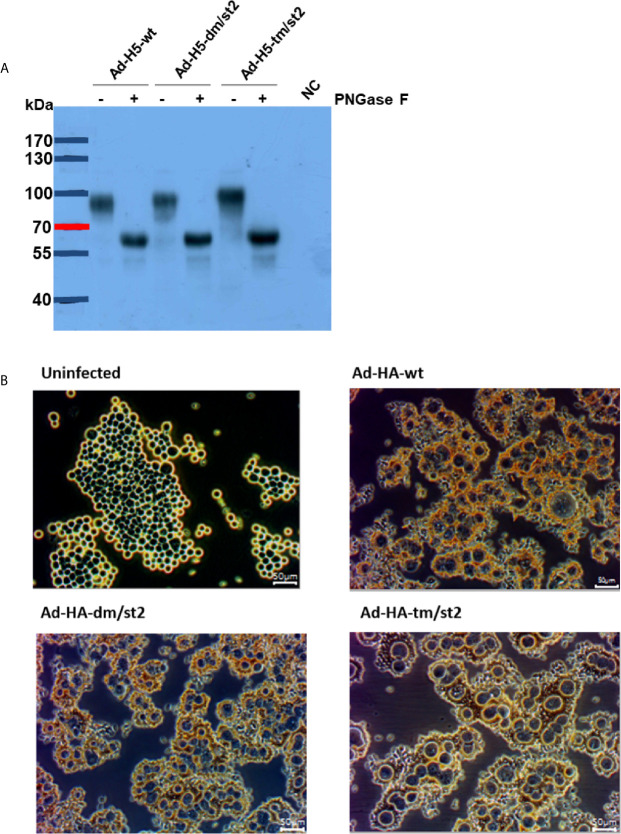
Characterizations of adenovirus vector encoding the full-length H5 gene with a combination of glycan-masking and glycan-unmasking HA antigen design. **(A)** WT and mutant H5 proteins expressed in adenovirus vector-infected 293A cells were confirmed by western blotting with anti-H5 antibody. **(B)** RBCs absorbed on adenovirus-infected cells were observed by microscopy.

Prime-boost immunizations provide an advantage by more effectively recalling immune responses to H5 antigens to elicit more potent neutralizing antibodies after rH5 booster immunizations. To further enhance the anti-H5N1 immunity, we previously reported using an adenovirus vector prime followed by an rH5 booster immunization regimen that resulted in approximately 0.5-log increased neutralizing antibody titers, as compared to two-dose rH5 regimen (16, 19). Therefore, in this study we investigated these two vaccination regimens, two-dose rH5 protein regimen or adenovirus vector-prime + rH5 protein-boost regimen, in parallel to test the immunogenicity elicited by the glycan-engineered H5 antigen, with the special interest on the stem-specific immunity. We further constructed three adenovirus vectors of Ad-H5-wt, Ad-H5-dm/st2 and Ad-H5-tm/st2 encoding the full-length genes of H5-wt, H5-dm/st2, and H5-tm/st2, respectively. These adenovirus vectors were obtained from transfected 293A cells and analyzed by western blotting for the expression of the full-length H5 protein (i.e. 100 kDa mol weight) in cell lysates separated by SDS-PAGE gels (**Figure 2A**). The 293A cells infected with these adenovirus vectors (Ad-H5-wt, Ad-H5-dm/st2, and Ad-H5-tm/st2) were all capable of inducing RBC hemagglutination in comparison to the uninfected control (**Figure 2B**).

### Immunizations by rH5 + rH5 or Ad-H5 (Prime) + rH5 (Boost) Regimen

**Figure 3:**
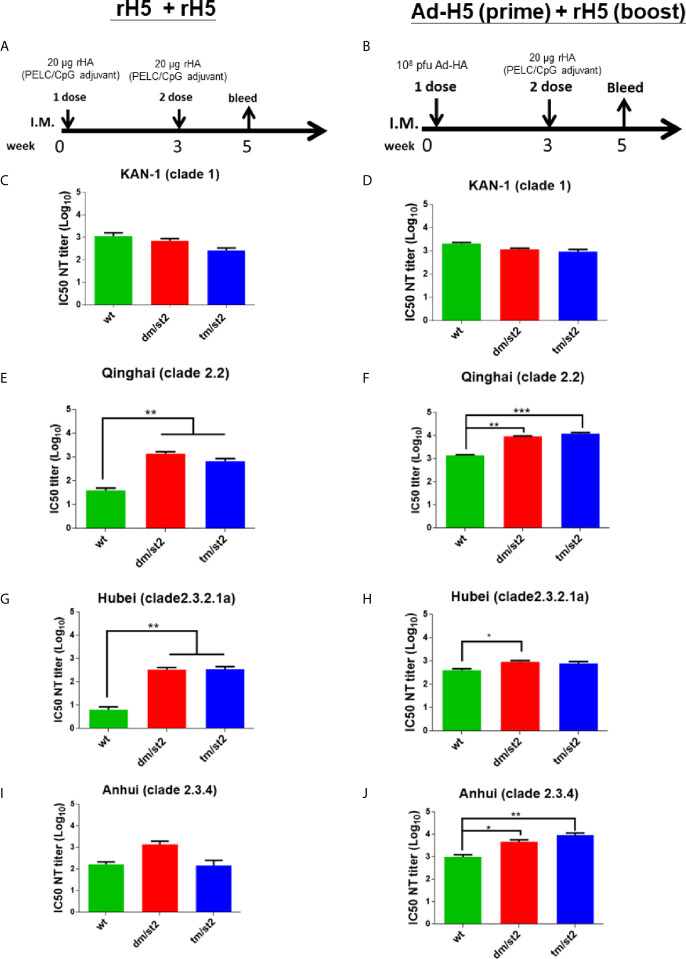
Neutralizing antibody titers against the homologous and heterologous H5N1 strain elicited by immunizations with two-dose rH5 protein or adenovirus vector-prime + rH5 protein-boost regimen. Groups of BALB/c mice were immunized by **(A)** rH5 + rH5: two-dose rH5 proteins with PELC/CpG adjuvant and **(B)** Ad-H5 (prime) + rH5 (boost): first-dose Ad-H5 and second dose rH5 with PELC/CpG adjuvant. Neutralizing antibody titers of IC50 values of rH5 + rH5 regimen against **(C)** KAN-1 (clade 1), **(E)** Qinhai (clade 2.2), **(G)** Hubei (clade 2.3.2.1a), and **(I)** Anhui (clades 2.3.4). IC50 values of Ad-H5 (prime) + rH5 (boost) regimen against **(D)** KAN-1 (clade 1), **(F)** Qinhai (clade 2.2), **(H)** Hubei (clade 2.3.2.1a), and **(J)** Anhui (2.3.4). Data were analyzed using one-way ANOVA. (*p < 0.05, **p < 0.01, and ***p < 0.001).

To investigate the antibody responses elicited by the combination of glycan-unmasking and glycan-masking HA antigens, we conducted the following two types of immunization regimens in groups of BALB/c mice through intramuscular injections: (i) two-dose 20 μg rH5 proteins with PELC/CpG adjuvant (rH5 + rH5) (**Figure 3A**) and (ii) first-dose 1 x 10^8^ pfu Ad-H5 and second dose 20 μg rH5 with PELC/CpG adjuvant (Ad-H5 (prime) + rH5 (boost)) (**Figure 3B**). We used the PELC/CpG adjuvant for rH5 immunization as we have previously demonstrated that rH5 proteins formulated with PELC/CpG can elicit more potent and cross-clade/subclade neutralizing antibodies and protective immunity against H5N1 virus infections (19). Results from antisera collected after the second immunization dose indicated that all the immunization groups (rH5-wt, rH5-dm/st2, or rH5-tm/st2) except the PBS control elicited a significant increase and a similar range of H5-specific IgG titers for the two-dose rH5 + rH5 regimen as determined by rH5 (KAN-1)-binding (**Figure S2A**) and H5N1 RG14 inactivated virus-coating ELISA (**Figure S2C**). Similar results were found for the Ad-H5 (prime) + rH5 (boost) immunization regimen with Ad-H5-wt + rH5-wt, Ad-H5-dm/st2 + rH5-dm/st2, and Ad-H5-tm/st2 + rH5-tm/st2 (**Figures S2B, S2D**). For neutralizing antibody titers, serially diluted antisera were pre-incubated with different clades and subclades of H5 pseudotyped particles (H5pp) (KAN-1 of clade 1, Qinhai of clade 2.2, Hubei of clade 2.3.2.1a, and Anhui of clade 2.3.4) to obtain the dose-dependent neutralization curves (**Figures S3A–H**) to calculate their corresponding IC50 values as the neutralizing antibody titers (**Figures 3C–J**). Our results indicated that for these two immunization regimens rH5 + rH5 and Ad-H5 (prime) + rH5 (boost) the IC50 values against the homologous KAN-1 H5N1 strain among the H5-wt, H5-dm/st2, and H5-tm/st2 immunized groups were similar (**Figures 3C, D**). However, two-dose immunizations with rH5-dm/st2 + rH5-tm/st2 resulted in significantly improved neutralizing antibody titers against two of the three heterologous H5pp strains of Qinhai (clade 2.2) and Hubei (clade 2.3.2.1a) (**Figures 3E, G, I**). Prime-boost immunizations with Ad-H5-dm/st2 + rH5-dm/st2 and Ad-H5-tm/st2 + rH5-tm/st2 also induced significantly higher titers of neutralizing antibodies against the heterologous Qinhai (clade 2.2) and Anhui (clade 2.3.4) (**Figures 3F, J**). Prime-boost immunizations with the Ad-H5-dm/st2 + rH5-dm/st2 group elicited significantly higher neutralizing antibody titers against the heterologous Hubei clade (clade 2.3.2.1a) (**Figure 3H**). Therefore, the use of a combination of double and triple glycan masking with a single glycan-unmasking HA antigen design (H5-dm/st2 and H5-dm/st2) can induce higher neutralizing antibody titers against different H5N1 clade/subclade viruses.

### Mapping HA Stem-Binding Antibodies in Antisera

**Figure 4:**
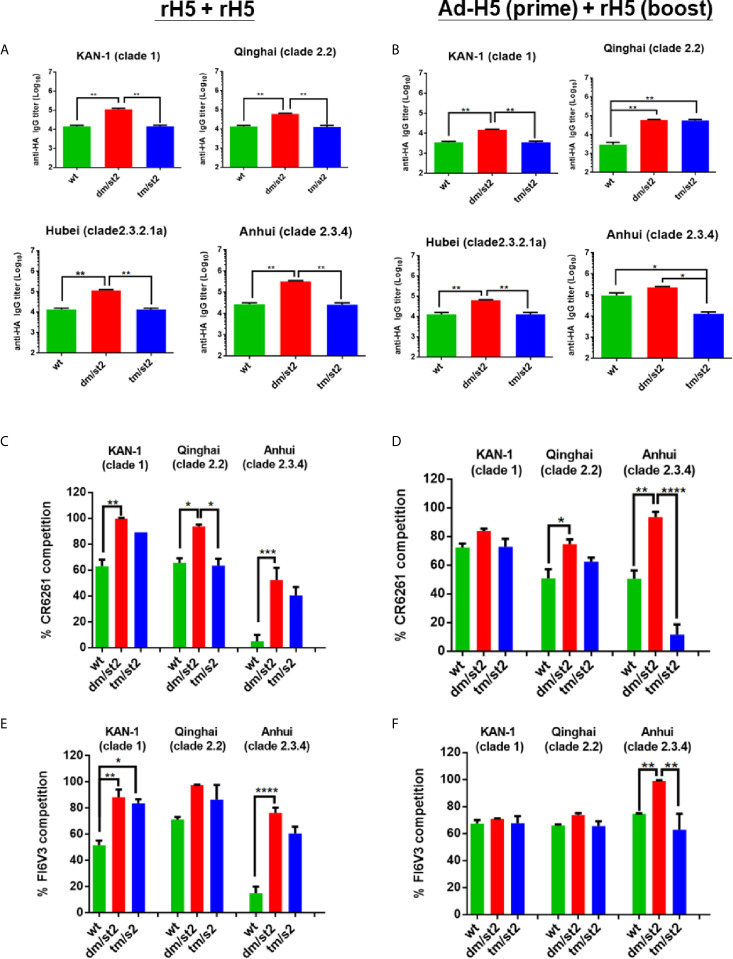
Mapping HA stem-binding antibodies in antisera. Antisera elicited by **(A)** rH5 + rH5 and **(B)** Ad-H5 (prime) + rH5 (boost) regimens were absorbed with Δstem-rH5 proteins and measured for HA-stem binding IgG titers using ELISA coated with different clade/subclade rH5 proteins. The HA stem-binding antibodies elicited by **(C)** rH5 + rH5 and **(D)** Ad-H5 (prime) + rH5 (boost) regimens were measured using mAb CR6261 competition. The HA stem-binding antibodies elicited by **(E)** rH5 + rH5 and **(F)** Ad-H5 (prime) + rH5 (boost) regimens were measured using mAb FI6v3 competition. Data were analyzed using one-way ANOVA. (*p < 0.05, **p < 0.01, ***p < 0.001, and ****p < 0.0001).

To investigate whether glycan masking with glycan-unmasking HA antigen design (H5-dm/st2 and H5-dm/st2) can refocus antibody responses to the HA stem-binding region, we mapped the HA binding region(s) for these polyclonal antisera with a mutant Δstem-rH5 protein that has been reported to abrogate stem-binding antibodies by the introduction of N-glycans in the conserved HA stem region (16, 18, 21). Antisera from each immunized group were absorbed with 40 μg/ml Δstem-rH5 proteins coupled on the Ni-nitrilotriacetic acid beads. The HA stem-binding IgG titers were determined from the absorbed sera by ELISAs coated with different clades of rH5 proteins. The results indicated that these two immunization regimens of rH5 + rH5 and Ad-H5 (prime) + rH5 (boost) for the H5-dm/st2 antigens compared to the H5-wt and H5-tm/st2 groups significantly increased the HA stem-binding antibodies to the homologous Kan-1 (clade 1), as well as three heterologous clades: Qinhai (clade 2.2), Hubei (clade 2.3.2.1a), and Anhui (clade 2.3.4) (**Figures 4A, B**). Therefore, the glycan-masking and glycan-unmasking H5-dm/st2 antigen elicited more HA stem-binding antibodies than the H5-wt and H5-tm/st2 antigens. These results were further confirmed by a competition assay against the Δstem-rH5 absorbed antisera using two well-known HA stem-binding broadly neutralizing mAb CR6261 for the group 1 subtype (28, 29) and mAb FI6v3 for both group 1 and 2 subtypes (21, 30). For the mAb CR6261 competition assay, two-dose rH5 immunization or Ad-H5 primed followed by rH5 booster immunization with the H5-dm/st2 antigens elicited higher titers of stem-binding antibodies against the homologous Kan-1 (clade 1) and the heterologous Qinhai (clade 2.2) and Anhui (clade 2.3.4) (**Figures 4C, D**). For the mAb FI6v3 competition assay, two-dose rH5-dm/st2 immunization compared to the H5-wt antigen elicited higher titers of HA stem-binding antibodies to the homologous Kan-1 (clade 1) and the heterologous Anhui (clade 2.3.4) (**Figure 4E**). Prime-boost immunizations by Ad-H5-dm/st2 + rH5-dm/st2 resulted in significantly increased stem-binding antibodies against the heterologous Anhui (clade 2.3.4) (**Figure 4F**). All of these results indicated that immunizations with the glycan-masking and glycan-unmasking H5-dm/st2 antigen elicited more HA stem-binding antibodies in antisera.

### Inhibition of HA Stem-Mediated Fusion Activity

**Figure 5:**
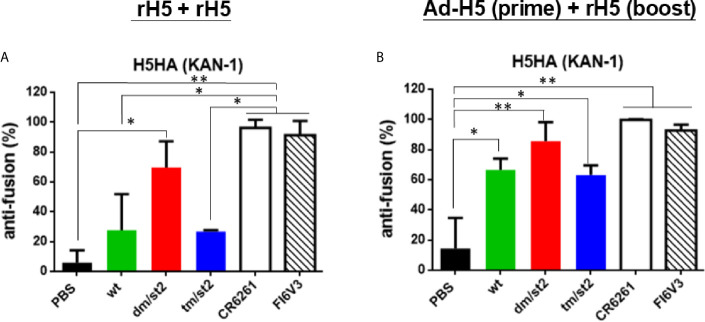
Inhibition of HA stem-mediated fusion activity. HA stem-mediated fusion inhibition activity was measured using a luciferase-based cell-cell fusion assay. Stem-binding mAbs CR6261 and FI6v3 were used as the positive controls. **(A)** HA stem-mediated fusion inhibition activity of antisera elicited by rH5 + rH5 regimen. **(B)** HA stem-mediated fusion inhibition activity of antisera elicited by Ad-H5 (prime) + rH5 (boost) regimen. Data were analyzed using one-way ANOVA. (*p < 0.05, and **p < 0.01).

To measure the HA stem-mediated fusion activity in antisera, we used a luciferase-based cell-cell fusion assay to determine the fusion inhibition activity as previously reported (18, 22, 31). The percentage of the HA-mediated membrane fusion between the donor and indicator cells was determined by the expression of luciferase in the co-cultured cells. Our data indicated that the H5-dm/st2 antigens enhanced fusion inhibition activity compared to the H5-wt and H5-tm/st2 groups by rH5 + rH5 two-dose immunizations (**Figure 5A**) and by Ad-H5-dm/st2 + rH5-dm/st2 prime-boost immunizations (**Figure 5B**). The HA stem-mediated cell-cell fusion activity was also completely blocked by the stem-binding mAbs CR6261 and FI6v3 (**Figures 5A, B**). Therefore, only the glycan-masking and glycan-unmasking H5-dm/st2 antigen elicited antisera with HA stem-mediated fusion inhibition activity.

### Protective Immunity Against Homologous and Heterologous H5N1 Virus Challenges

**Figure 6:**
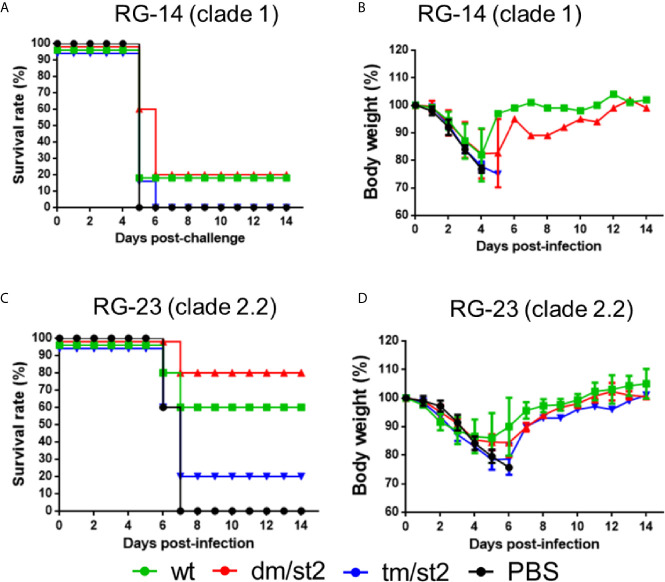
Protective immunity against homologous and heterologous H5N1 virus challenges. Four groups of mice immunized with rH5 + rH5 regimen, or alone with the PBS control were challenged with 10-fold of the 50% mouse lethal dose (MLD50) of the homologous RG14 (clade 1) or the heterologous RG‐23 (clade 2.2) H5N1 viruses. **(A)** Survival rates of mice challenged with RG14 (clade 1) H5N1 virus. **(B)** Body weights of mice challenged with RG14 (clade 1) H5N1 virus. **(C)** Survival rates of mice challenged with RG‐23 (clade 2.2) H5N1 virus. **(D)** Body weights of mice challenged with RG‐23 (clade 2.2) H5N1 virus.

To determine protective immunity against the homologous and heterologous H5N1 virus clade/subclade viruses, four groups of mice immunized with the rH5 + rH5 regimen, or alone with the PBS control were challenged with 10-fold of the 50% mouse lethal dose (MLD50) of the homologous RG14 (clade 1) or the heterologous RG‐23 (clade 2.2) H5N1 viruses. For the homologous H5N1 virus challenges, the glycan-masking and glycan-unmasking rH5-dm/st2 and the rH5-wt had a 20% survival rate with a full recovery of body weight loss 14 days after virus challenge compared to the 0% survival rate for the rH5-tm/st2 and PBS immunized groups (**Figures 6A, B**). For the heterologous RG-23 virus challenge, the survival rate for the rH5-dm/st2-immunized group was 80%, followed by 60% for the rH5-wt immunized group, 20% for the rH5-tm/st2-immunized group, and 0% for the PBS-immunized group (**Figure 6C**). Body weight loss and recovery did not show significant differences among the rH5-wt, rH5-dm/str, and rHA-tm/st2 groups (**Figure 6D**). Therefore, only the glycan-masking and glycan-unmasking H5-dm/st2 antigen provided improved protection against heterologous clade virus challenges.

### Generation and Characterization of H5N1 RG Viruses Containing the H5-wt or H5-dm/st2 Gene

**Figure 7:**
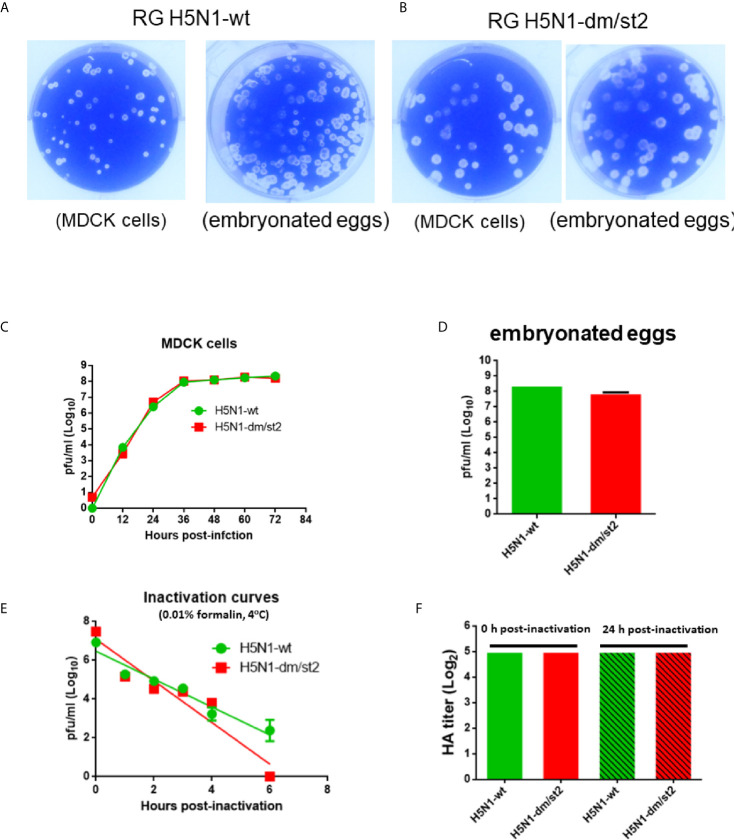
Generation and characterization of H5N1 RG viruses containing the H5-wt or H5-dm/st2 gene. **(A)** Viral plaques of RG H5N1-wt virus propagated in MDCK cells and embryonated chicken eggs. **(B)** Viral plaques of RG H5N1-dm/st2 virus propagated in MDCK cells and embryonated chicken eggs. **(C)** Propagation kinetics of RG H5N1-wt and H5N1-dm/st2 RG viruses in MDCK cells. **(D)** The peak titers of RG H5N1-wt and H5N1-dm/st2 RG viruses propagated in embryonated chicken eggs. **(E)** Formalin inactivation kinetics of RG H5N1-wt and H5N1-dm/st2 RG viruses. **(F)** The HA contents of RG H5N1-wt and RG H5N1-dm/st2 viruses before and after formalin inactivation.

**Figure 8:**
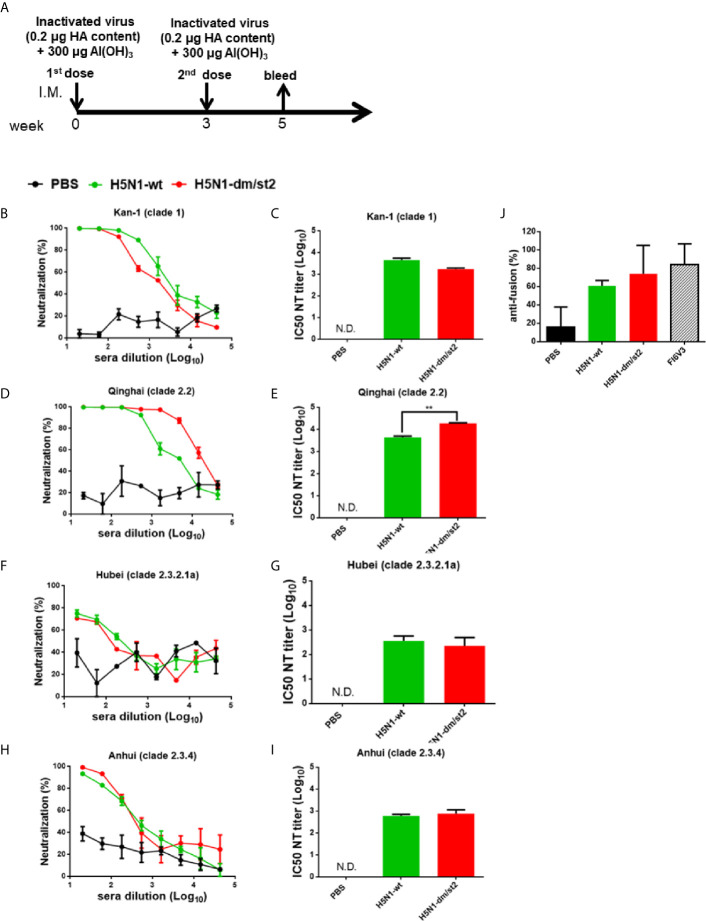
Immunizations with inactivated RG H5N1-wt and RG H5N1-dm/st2 viruses formulated with alum adjuvant. **(A)** Inactivated RG H5N1-wt and RG H5N1-dm/st2 viruses were immunized intramuscularly for two doses in a three-week interval. Each dose contained 0.2 μg HA content formulated with 300 μg alumn adjuvant. Serum samples were bled at week 5 and measured for virus-neutralizing antibodies against various clades/subclades of H5N1 viruses. Neutralization curves and their corresponding IC50 titers were plotted against **(B, C)** KAN-1(clade 1), **(D, E)** Qinghai (clade 2.2), **(F, G)** Hubei (clade 2.3.2.1a), and **(H, I)** Anhui (clade 2.3.4). **(J)** HA stem-mediated fusion inhibition activity of antisera were determined using a luciferase-based cell-cell fusion assay. Stem-binding mAb FI6v3 was used as a positive control. Data were analyzed using one-way ANOVA. (**p < 0.01).

The PR8 [A/Puerto Rico/8/1934 (H1N1)] 8 plasmid-based reverse genetic system was used to generate H5N1 RG viruses. The PR8 HA gene replaced with the Kan-1 H5 or H5-dm/st2 mutant gene (the HA cleavage site changed from RRRKK to T) and the NA gene of A/Viet Nam/1203/2004(H5N1) were transfected into 293 cells with six plasmids of other PR8 genes to obtain RG H5N1-wt and RG H5N1-dm/st2 RG viruses. Both RG H5N1-wt and RG H5N1-dm/st2 viruses were effectively propagated in MDCK cells and embryonated chicken eggs as shown by visible viral plaques (**Figures 7A, B**). No differences were observed in the propagation kinetics in MDCK cells and the peak titers in embryonated chicken eggs between RG H5N1-wt and RG H5N1-dm/st2 viruses (**Figures 7C, D**). Formalin inactivation showed a faster kinetics for the RG H5N1-dm/st2 virus compared to the RG H5N1-wt virus (**Figure 7E**). The HA contents of both RG H5N1-wt and RG H5N1-dm/st2 viruses before and after formalin inactivation remained approximately the same values (**Figure 7F**). Immunization with the inactivated RG H5N1-wt and RG H5N1-dm/st2 viruses were further conducted in BALB/c mice with 0.2 μg HA dose plus alum adjuvant for two doses and serum samples were collected 2 weeks after the second dose (**Figure 8A**). Dose-dependent neutralization curves were obtained by serially diluting sera pre-incubated with H5pp of different clade/subclade (**Figures 8B, D, F, H**). The results indicated that the inactivated RG H5N1-dm/st2 virus compared to the inactivated RG H5N1-wt virus elicited a significantly higher neutralization antibody titer against the heterologous Qinhai (clade 2.2) H5N1 virus (**Figure 8E**) and retained similar titers against the homologous Kan-1 (clade 1) and the two other heterologous Hubei (clade 2.3.2.1a) and Anhui (clade 2.3.4) viruses (**Figures 8C, G, I**). We also used the luciferase-based cell-cell fusion assay to examine the inhibition of HA stem-mediated fusion activity in antisera. The results showed that the inactivated RG H5N1-dm/st2 virus enhanced fusion inhibition activity compared to the inactivated RG H5N1-wt virus (**Figure 8J**). Therefore, the inactivated RG H5N1-dm/st2 virus can elicit a higher titer of cross-clade neutralizing antibodies with more anti-fusion antibodies against different H5N1 clades.

## Discussion

As the WHO has recommended 32 candidate vaccine viruses to meet H5N1 vaccine preparation (9), it is important to develop broader cross-clade H5N1 vaccines. In this study we investigated two antigen designs combining glycan-masking and glycan unmasking H5 (H5-dm/st2 and H5-tm/st2) using three types of immunization regimens: (i) two-dose protein immunization (rH5 + rH5), (ii) adenovirus vector priming, followed by rH5 protein immunization [Ad-H5 (prime) + rH5 (boost)], and (iii) two-dose inactivated H5N1 virus immunization. The results indicated that only the glycan-masking and glycan-unmasking H5-dm/st2 antigen design was capable of eliciting a broader range of neutralizing antibodies against different H5N1 virus strains and improved protection against the heterologous H5N1 virus challenges. The use of a combination of glycan-masking and glycan-unmasking antigen design of H5-dm/st2 but not H5-tm/st2 resulted in refocusing anti-HA antibody responses to the more conserved stem-binding region for anti-fusion antibodies.

Using a combination of glycan-masking and glycan-unmasking antigen design strategies, we obtained soluble proteins of rH5, rH5-dm/st2, and rH5-tm/st2 and examined the RBC agglutination and fetuin-binding properties *in vitro*. Results indicated that the purified rH5-dm/st2 proteins had a 3-fold higher titer for *in vitro* RBC agglutination than the purified rH5-wt proteins (**Figure 1C**). In contrast, the purified rH5-tm/st2 proteins abolished RBC agglutination. The results were again confirmed using fetuin-binding assay (**Figure 1D**). However, three adenovirus vectors of Ad-H5-wt, Ad-H5-dm/st2 and Ad-H5-tm/st2 encoding the full-length HA gene were all capable of inducing RBC hemagglutination in comparison to the uninfected control (**Figure 2B**). Since these three rH5 proteins were constructed using a GCN4-pII leucine zipper sequence for trimerization to improve the stability, it is likely that the addition of two N-linked glycosylation motifs on H5 residues 127 and 138, which are close to the 130-loop of the receptor binding sites (14), can retain the overall-folded rH5 proteins for RBC agglutination and fetuin-binding properties. But the additional N-glycan added on the H5 residue 83, which is near the inner monomeric HA interface (32), may thus disrupt the rH5 protein structure for RBC hemagglutination. In contrast, the adenovirus vectors encoding the full-length HA with the native transmembrane domain can retain the authentic RBC hemagglutination epitopes for the H5-tm/st2 mutation.

Two immunization regimens, (i) two-dose rH5 + rH5 and (ii) Ad-H5 (prime) + rH5 (boost), were first examined in parallel for the elicitation of cross-clade/subclade neutralizing antibodies and protection against the heterologous H5N1 clade/subclade viruses. The results indicate that the use of H5-dm/st2 antigen elicited higher titers of cross-clade/subclade neutralizing antibodies against three heterologous virus strains. The results were in concordance with the increase in HA stem-binding antibodies using pre-absorbed antisera with Δstem-rH5 protein-coupled beads (**Figures 4A, B**) and the competition with two stem-specific neutralizing mAbs CR6261 and FI6v3 (**Figures 4C–F**). Therefore, immunizations with the H5-dm/st2 antigen for both rH5 + rH5 and Ad-H5 (prime) + rH5 (boost) regimens resulted in increasing a significant fraction of serum antibodies that could redirect the antibody responses to the H5 stem region. The increased amounts of HA stem-specific antibodies elicited by H5-dm/st2 immunizations were correlated with the increased inhibition of the HA stem-mediated cell-cell fusion (**Figure 5**), which is triggered by the pH-dependent conformational change of the HA trimer to mediate cell-cell fusion of the viral membrane to the host cell endosome membrane (10). One of the universal influenza vaccine design strategies focused on eliciting HA stem-specific antibodies, because the HA stem regions are highly conserved across different influenza virus strains and subtypes (33–37). However, the antisera obtained by H5-dm/st2 immunizations in this study did not induce cross-neutralizing antibodies against other influenza subtypes such as pH1N1 and H3N2 (**Figure S4**). The chimeric HA chimeric HA vaccine with consensus H5 as globular head and consensus H1 as stem was recently shown to elicit broadly protective CD4+ and CD8+ T cell responses against divergent strains of H1N1 and H5N1 viruses (38). Further engineering the H5-dm/st2 antigen replaced with the H1 stem domain may provide a universal protection against different influenza subtypes.

The glycan-masking and glycan-unmasking antigen design for H5-dm/st2 was finally translated to generate RG viruses using PR8-based reverse genetics technology. Both RG H5N1-dm/st2 and RG H5N1-wt viruses were rescued and effectively propagated in MDCK cells and embryonated chicken eggs (**Figures 7A–D**). Although the RG H5N1-dm/st2 virus displayed a faster formalin inactivation kinetics compared to that of the RG H5N1-wt virus, it retained a similar range of HA titers before and after formalin inactivation (**Figure 7E**). We also measure the NA activity for the RG H5N1-dm/st2 and H5N1-wt viruses before and after formalin inactivation, and the results were also the same (data not shown). Immunizations with the inactivated RG H5N1-dm/st2 virus compared to the inactivated RG H5N1-wt virus at 0.2 μg HA dose were found to elicit a significantly higher titer of cross-clade neutralizing antibodies against the heterologous Qinghai (clade 2.2) H5N1 viruses (**Figure 8**). The inactivated H5N1-dm/st2 RG virus was also shown to elicit more of the HA-stem directed antibodies for anti-fusion activity (**Figure 8J**). The H5N1-dm/st2 RG virus with the glycan-masking on the globular head and the glycan-unmasking on the stem region can be used for further development of cross-protective H5N1 vaccines.

## Data Availability Statement

The raw data supporting the conclusions of this article will be made available by the authors, without undue reservation.

## Ethics Statement

The animal study was reviewed and approved by the Laboratory Animal Center of National Tsing Hua University (NTHU). Animal use protocols were reviewed and approved by the NTHU Institutional Animal Care and Use Committee (approval no. 108044).

## Author Contributions

T-HC, C-CC, and S-CW designed the research. T-HC, Y-LY, and J-TJ performed the experiments and analyzed the data. T-HC, Y-LY, and S-CW wrote the manuscript and created the figures. All authors contributed to the article and approved the submitted version.

## Funding

This work was supported by the Ministry of Science and Technology, Taiwan (MOST109-2313-B-007-001-MY2, MOST109-2327-B-007-003) and Hsinchu MacKay Memorial Hospital (MMH-HB-10906).

## Conflict of Interest

The authors declare that the research was conducted in the absence of any commercial or financial relationships that could be construed as a potential conflict of interest.

## Supplementary Material

Supplementary Figure 1 Structural diagrams and site-specific glycan-masking and glycan-unmasking antigens. Amino acid sequences for **(A)** rH5-dm/st2 (^127^NSS+^138^NGT+^484^AGT) and **(B)** rH5-tm/st2 (^83^NSS+^127^NSS+^138^NGT+^484^AGT). The mutant sites are displayed by arrows. Three-dimensional structural models for **(C)** rH5-dm/st2 and **(D)** rH5-tm/st2 (PyMol modeling software; PDB ID: 2IBX). Gray color, HA trimeric structure; yellow, globular head; cyan, stem region; magenta, glycan-masking mutant sites; red, glycan-unmasking mutant site.

Supplementary Figure 2 IgG antibody titers against homologous H5N1 strain were elicited by immunizations with two-dose rH5 protein or adenovirus vector-prime + rH5 protein-boost regimen. Groups of BALB/c mice were immunized by rH5 + rH5: two-dose rH5 proteins with PELC/CpG adjuvant and Ad-H5 (prime) + rH5 (boost): first-dose Ad-H5 and second dose rH5 with PELC/CpG adjuvant. The H5-specific IgG titers as determined by rH5-coating ELISA **(A, B)** and H5N1 RG14 inactivated virus-coating ELISA **(C, D)**.

Supplementary Figure 3 Serum neutralization curves against the homologous and heterologous H5N1 viruses. Neutralization curves of antisera elicited by the rH5 + rH5 regimen against **(A)** KAN-1 (clade 1), **(B)** Qinhai (clade 2.2), **(C)** Hubei (clade 2.3.2.1a), and **(D)** Anhui (clades 2.3.4). Neutralization curves of antisera elicited by the Ad-H5 (prime) + rH5 (boost) regimen against **(E)** KAN-1 (clade 1), **(F)** Qinhai (clade 2.2), **(G)** Hubei (clade 2.3.2.1a), and **(H)** Anhui (2.3.4).

Supplementary Figure 4 Antisera against the heterosubtypic pH1N1 (A/California/04/2009) and H3N2 (A/Udorn/307/1972) viruses. Immunization with rH5 + rH5 regimen: **(A)** hemagglutinin inhibition against pH1N1, **(B)** hemagglutinin inhibition against H3N2, **(C)** PRNT neutralization curves against pH1N1, **(D)** PRNT neutralization curves against H3N2; Immunization with Ad-H5 (prime) + rH5 (boost) regimen: **(E)** hemagglutinin inhibition against pH1N1, **(F)** hemagglutinin inhibition against H3N2, **(G)** PRNT neutralization curves against pH1N1, **(H)** PRNT neutralization curves against H3N2.

## References

[1] Tran TH Nguyen TL Nguyen TD Luong TS Pham PM Nguyen V. Avian Influenza A (H5N1) in 10 Patients in Vietnam. N Engl J Med (2004) 350(12):1179–88. 10.1056/NEJMoa040419 14985470

[2] de Jong MD Simmons CP Thanh TT Hien VM Smith GJ Chau TN. Fatal Outcome of Human Influenza A (H5N1) is Associated With High Viral Load and Hypercytokinemia. Nat Med (2006) 12(10):1203–7. 10.1038/nm1477 PMC4333202 16964257

[3] Wang H Feng Z Shu Y Yu H Zhou L Zu R. Probable Limited Person-to-Person Transmission of Highly Pathogenic Avian Influenza A (H5N1) Virus in China. Lancet (2008) 371(9622):1427–34. 10.1016/S0140-6736(08)60493-6 18400288

[4] WHO. Cumulative Number of Confirmed Human Cases for Avian Influenza A (H5N1) Reported to WHO, 2003-2019. Available at: https://www.who.int/influenza/human_animal_interface/2019_06_24_tableH5N1.pdf?ua=1.

[5] Murakami S Iwasa A Iwatsuki-Horimoto K Ito M Kiso M Kida H. Cross-Clade Protective Immunity of H5N1 Influenza Vaccines in a Mouse Model. Vaccine (2008) 26(50):6398–404. 10.1016/j.vaccine.2008.08.053 PMC2626625 18804131

[6] Smith GJ Donis RO World Health Organization/World Organisation for Animal HF. Nomenclature Updates Resulting From the Evolution of Avian Influenza A(H5) Virus Clades 2.1.3.2a, 2.2.1, and 2.3.4 During 2013-2014. Influenza Other Respir Viruses (2015) 9(5):271–6. 10.1111/irv.12324 PMC4548997 25966311

[7] Watanabe Y Ibrahim MS Ellakany HF Kawashita N Daidoji T Takagi T. Antigenic Analysis of Highly Pathogenic Avian Influenza Virus H5N1 Sublineages Co-Circulating in Egypt. J Gen Virol (2012) 93(Pt 10):2215–26. 10.1099/vir.0.044032-0 22791605

[8] Lee DH Torchetti MK Winker K Ip HS Song CS Swayne DE. Intercontinental Spread of Asian-Origin H5N8 to North America Through Beringia by Migratory Birds. J Virol (2015) 89(12):6521–4. 10.1128/JVI.00728-15 PMC4474297 25855748

[9] WHO. Summary of Status of Development and Availability of A(H5N1) Candidate Vaccine Viruses and Potency Testing Reagents. Updated 12 October 2018. Available at: https://www.who.int/influenza/vaccines/virus/candidates_reagents/summary_a_h5n1_cvv_sh19_20181012.pdf?ua=1.

[10] Skehel JJ Wiley DC. Receptor Binding and Membrane Fusion in Virus Entry: The Influenza Hemagglutinin. Annu Rev Biochem (2000) 69:531–69. 10.1146/annurev.biochem.69.1.531 10966468

[11] Krammer F Palese P. Influenza Virus Hemagglutinin Stalk-Based Antibodies and Vaccines. Curr Opin Virol (2013) 3(5):521–30. 10.1016/j.coviro.2013.07.007 PMC3804342 23978327

[12] Steel J Lowen AC Wang TT Yondola M Gao Q Haye K. Influenza Virus Vaccine Based on the Conserved Hemagglutinin Stalk Domain. mBio (2010) 1(1):e00018–10. 10.1128/mBio.00018-10 PMC2912658 20689752

[13] Das SR Puigbo P Hensley SE Hurt DE Bennink JR Yewdell JW. Glycosylation Focuses Sequence Variation in the Influenza A Virus H1 Hemagglutinin Globular Domain. PloS Pathog (2010) 6(11):e1001211. 10.1371/journal.ppat.1001211 21124818 PMC2991263

[14] Medina RA Stertz S Manicassamy B Zimmermann P Sun X Albrecht RA. Glycosylations in the Globular Head of the Hemagglutinin Protein Modulate the Virulence and Antigenic Properties of the H1N1 Influenza Viruses. Sci Transl Med (2013) 5(187):187ra70. 10.1126/scitranslmed.3005996 PMC3940933 23720581

[15] Lin SC Lin YF Chong P Wu SC. Broader Neutralizing Antibodies Against H5N1 Viruses Using Prime-Boost Immunization of Hyperglycosylated Hemagglutinin DNA and Virus-Like Particles. PloS One (2012) 7(6):e39075. 10.1371/journal.pone.0039075 22720032 PMC3374787

[16] Lin SC Liu WC Jan JT Wu SC. Glycan Masking of Hemagglutinin for Adenovirus Vector and Recombinant Protein Immunizations Elicits Broadly Neutralizing Antibodies Against H5N1 Avian Influenza Viruses. PloS One (2014) 9(3):e92822. 10.1371/journal.pone.0092822 24671139 PMC3966833

[17] Chen TH Liu WC Lin CY Liu CC Jan JT Spearman M. Glycan-Masking Hemagglutinin Antigens From Stable CHO Cell Clones for H5N1 Avian Influenza Vaccine Development. Biotechnol Bioeng (2019) 116(3):598–609. 10.1002/bit.26810 30080931

[18] Liu WC Jan JT Huang YJ Chen TH Wu SC. Unmasking Stem-Specific Neutralizing Epitopes by Abolishing N-Linked Glycosylation Sites of Influenza Virus Hemagglutinin Proteins for Vaccine Design. J Virol (2016) 90(19):8496–508. 10.1128/JVI.00880-16 PMC5021406 27440889

[19] Lin SC Huang MH Tsou PC Huang LM Chong P Wu SC. Recombinant Trimeric HA Protein Immunogenicity of H5N1 Avian Influenza Viruses and Their Combined Use With Inactivated or Adenovirus Vaccines. PloS One (2011) 6(5):e20052. 10.1371/journal.pone.0020052 21655326 PMC3104987

[20] Nefkens I Garcia JM Ling CS Lagarde N Nicholls J Tang DJ. Hemagglutinin Pseudotyped Lentiviral Particles: Characterization of a New Method for Avian H5N1 Influenza Sero-Diagnosis. J Clin Virol (2007) 39(1):27–33. 10.1016/j.jcv.2007.02.005 17409017

[21] Wei CJ Boyington JC McTamney PM Kong WP Pearce MB Xu L. Induction of Broadly Neutralizing H1N1 Influenza Antibodies by Vaccination. Science (2010) 329(5995):1060–4. 10.1126/science.1192517 20647428

[22] Takikawa S Ishii K Aizaki H Suzuki T Asakura H Matsuura Y. Cell Fusion Activity of Hepatitis C Virus Envelope Proteins. J Virol (2000) 74(11):5066–74. 10.1128/JVI.74.11.5066-5074.2000 PMC110858 10799580

[23] Su Y Yang H Zhang B Qi X Tien P. A Dual Reporter Gene Based System to Quantitate the Cell Fusion of Avian Influenza Virus H5N1. Biotechnol Lett (2008) 30(1):73–9. 10.1007/s10529-007-9521-4 17823774

[24] Hoffmann E Neumann G Kawaoka Y Hobom G Webster RG. A DNA Transfection System for Generation of Influenza A Virus From Eight Plasmids. Proc Natl Acad Sci USA (2000) 97(11):6108–13. 10.1073/pnas.100133697 PMC18566 10801978

[25] Suguitan AL Jr. McAuliffe J Mills KL Jin H Duke G Lu B. Live, Attenuated Influenza A H5N1 Candidate Vaccines Provide Broad Cross-Protection in Mice and Ferrets. PloS Med (2006) 3(9):e360. 10.1371/journal.pmed.0030360 16968127 PMC1564176

[26] Tzeng TT Lai CC Weng TC Cyue MH Tsai SY Tseng YF. The Stability and Immunogenicity of Inactivated MDCK Cell-Derived Influenza H7N9 Viruses. Vaccine (2019) 37(47):7117–22. 10.1016/j.vaccine.2019.03.024 31383484

[27] Broecker F Zheng A Suntronwong N Sun W Bailey MJ Krammer F. Extending the Stalk Enhances Immunogenicity of the Influenza Virus Neuraminidase. J Virol (2019) 93(18):e00840–19. 10.1128/JVI.00840-19 PMC6714795 31375573

[28] Throsby M van den Brink E Jongeneelen M Poon LL Alard P Cornelissen L. Heterosubtypic Neutralizing Monoclonal Antibodies Cross-Protective Against H5N1 and H1N1 Recovered From Human IgM+ Memory B Cells. PloS One (2008) 3(12):e3942. 10.1371/journal.pone.0003942 19079604 PMC2596486

[29] Ekiert DC Bhabha G Elsliger MA Friesen RH Jongeneelen M Throsby M. Antibody Recognition of a Highly Conserved Influenza Virus Epitope. Science (2009) 324(5924):246–51. 10.1126/science.1171491 PMC2758658 19251591

[30] Corti D Voss J Gamblin SJ Codoni G Macagno A Jarrossay D. A Neutralizing Antibody Selected From Plasma Cells That Binds to Group 1 and Group 2 Influenza A Hemagglutinins. Science (2011) 333(6044):850–6. 10.1126/science.1205669 21798894

[31] Lin HH Huang LM Wu SC. A Quantitative Luciferase-Based Cell-Cell Fusion Assay to Measure Four-Serotype Dengue Virus E Protein-Triggered Membrane Fusion. Hum Vaccines Immunother (2020) 16(9):2176–82. 10.1080/21645515.2020.1748989 PMC7553686 32530355

[32] Shih AC Hsiao TC Ho MS Li WH. Simultaneous Amino Acid Substitutions at Antigenic Sites Drive Influenza A Hemagglutinin Evolution. Proc Natl Acad Sci USA (2007) 104(15):6283–8. 10.1073/pnas.0701396104 PMC1851070 17395716

[33] Yassine HM Boyington JC McTamney PM Wei CJ Kanekiyo M Kong WP. Hemagglutinin-Stem Nanoparticles Generate Heterosubtypic Influenza Protection. Nat Med (2015) 21(9):1065–70. 10.1038/nm.3927 26301691

[34] Impagliazzo A Milder F Kuipers H Wagner MV Zhu XY Hoffman RMB. A Stable Trimeric Influenza Hemagglutinin Stem as a Broadly Protective Immunogen. Science (2015) 349(6254):1301–6. 10.1126/science.aac7263 26303961

[35] Coughlan L Palese P. Overcoming Barriers in the Path to a Universal Influenza Virus Vaccine. Cell Host Microbe (2018) 24(1):18–24. 10.1016/j.chom.2018.06.016 30001520

[36] Wu NC Yamayoshi S Ito M Uraki R Kawaoka Y Wilson A. Recurring and Adaptable Binding Motifs in Broadly Neutralizing Antibodies to Influenza Virus Are Encoded on the D3-9 Segment of the Ig Gene. Cell Host Microbe (2018) 24(4):569–578 e4. 10.1016/j.chom.2018.09.010 30308159 PMC6327842

[37] Estrada LD Schultz-Cherry S. Development of a Universal Influenza Vaccine. J Immunol (2019) 202(2):392–8. 10.4049/jimmunol.1801054 PMC6327971 30617121

[38] Liao HY Wang SC Ko YA Lin KI Ma C Cheng TR. Chimeric Hemagglutinin Vaccine Elicits Broadly Protective CD4 and CD8 T Cell Responses Against Multiple Influenza Strains and Subtypes. Proc Natl Acad Sci USA (2020) 117(30):17757–63. 10.1073/pnas.2004783117 PMC7395492 32669430

