# Supplementary figures and images for: Site-Specific Glycan-Masking/Unmasking Hemagglutinin Antigen Design to Elicit Broadly Neutralizing and Stem-Binding Antibodies Against Highly Pathogenic Avian Influenza H5N1 Virus Infections
Source: Front Immunol. 2021 Jul 14;12:692700. doi: 10.3389/fimmu.2021.692700 (PMC8317614; doi:10.3389/fimmu.2021.692700)

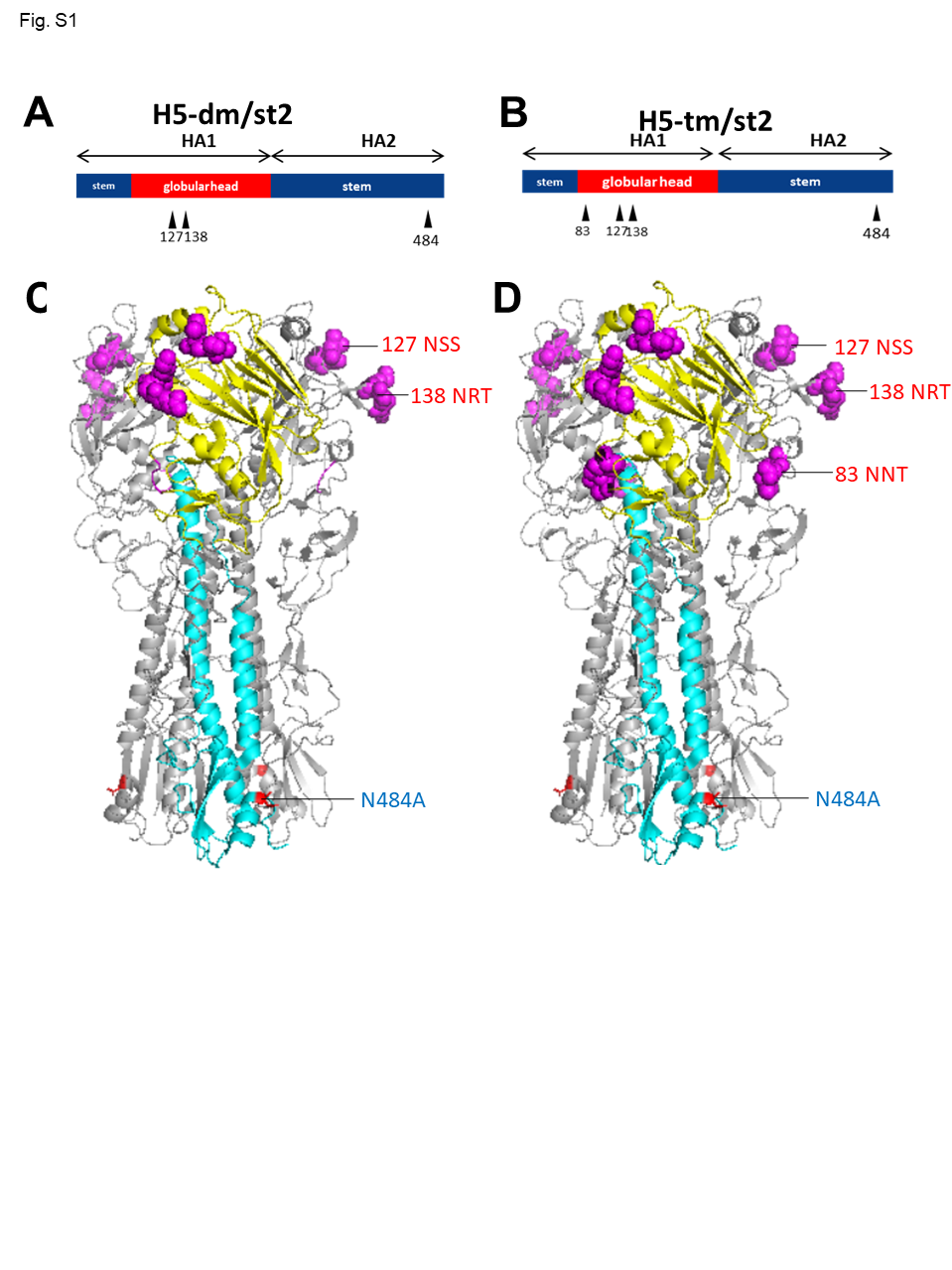

Supplement: Supplementary Figure 1 — Structural diagrams and site-specific glycan-masking and glycan-unmasking antigens. Amino acid sequences for (A) rH5-dm/st2 (127NSS+138NGT+484AGT) and (B) rH5-tm/st2 (83NSS+127NSS+138NGT+484AGT). The mutant sites are displayed by arrows. Three-dimensional structural models for (C) rH5-dm/st2 and (D) rH5-tm/st2 (PyMol modeling software; PDB ID: 2IBX). Gray color, HA trimeric structure; yellow, globular head; cyan, stem region; magenta, glycan-masking mutant sites; red, glycan-unmasking mutant site. [file Image_1.tif]

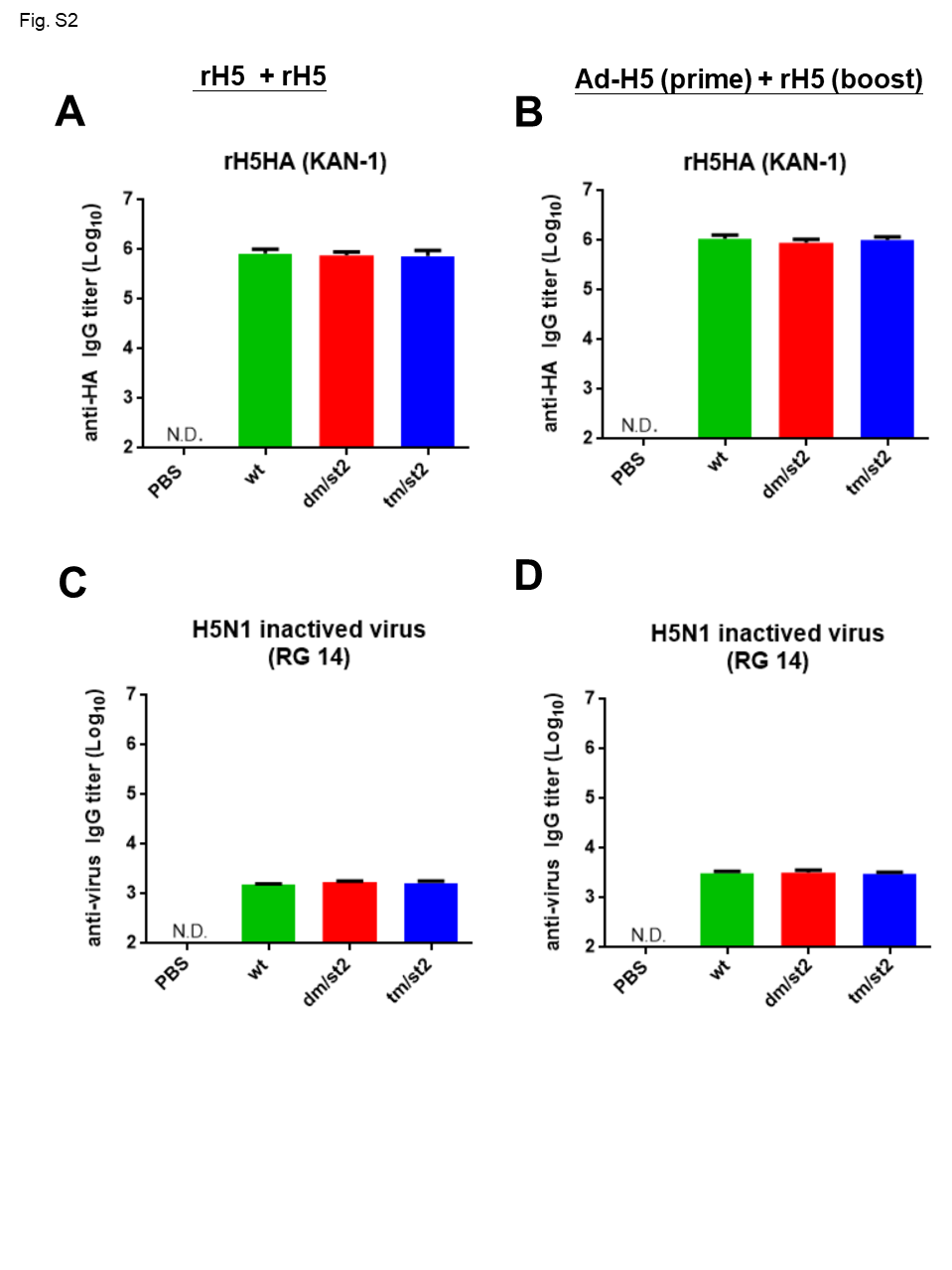

Supplement: Supplementary Figure 2 — IgG antibody titers against homologous H5N1 strain were elicited by immunizations with two-dose rH5 protein or adenovirus vector-prime + rH5 protein-boost regimen. Groups of BALB/c mice were immunized by rH5 + rH5: two-dose rH5 proteins with PELC/CpG adjuvant and Ad-H5 (prime) + rH5 (boost): first-dose Ad-H5 and second dose rH5 with PELC/CpG adjuvant. The H5-specific IgG titers as determined by rH5-coating ELISA (A, B) and H5N1 RG14 inactivated virus-coating ELISA (C, D). [file Image_2.tif]

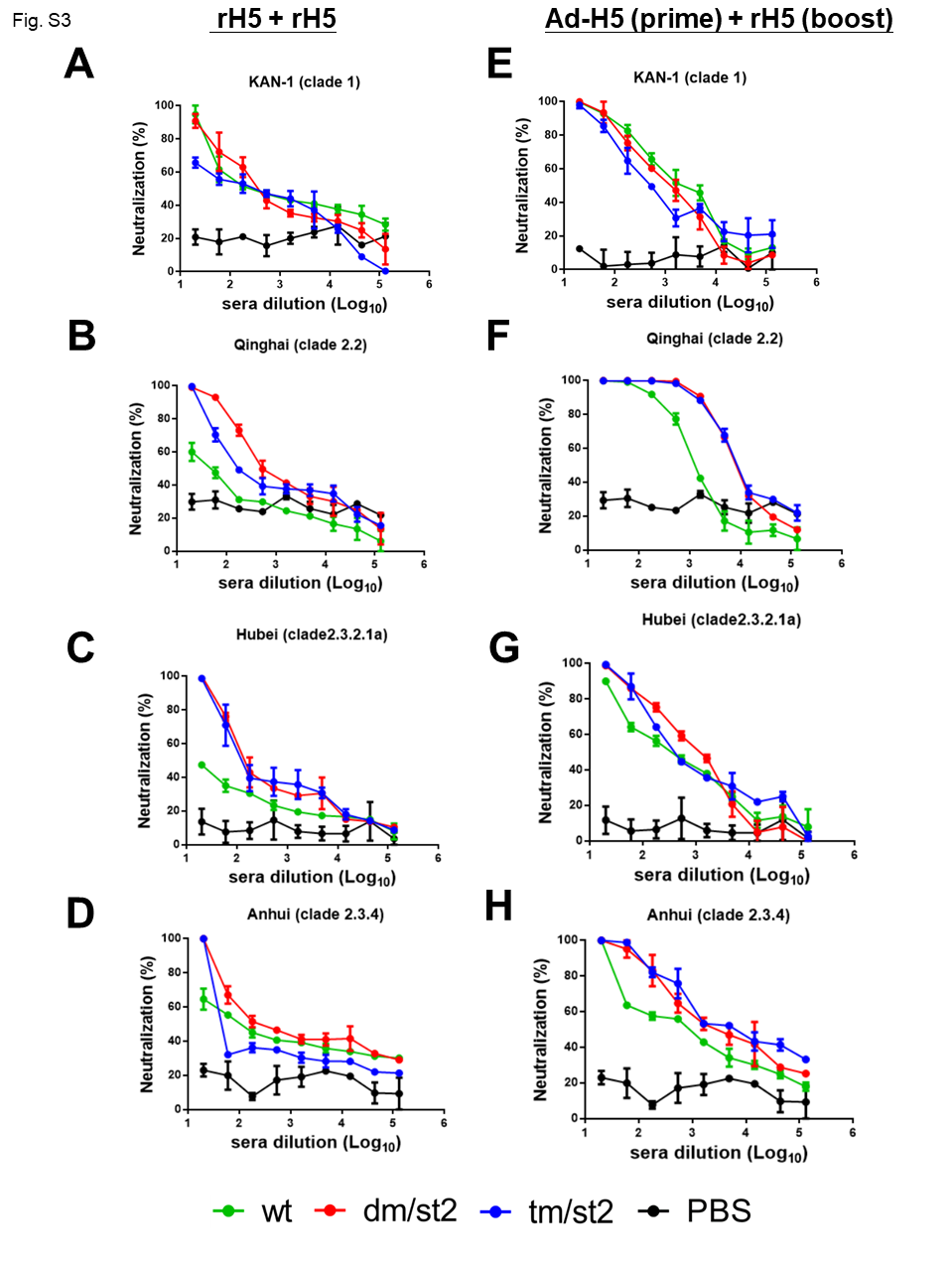

Supplement: Supplementary Figure 3 — Serum neutralization curves against the homologous and heterologous H5N1 viruses. Neutralization curves of antisera elicited by the rH5 + rH5 regimen against (A) KAN-1 (clade 1), (B) Qinhai (clade 2.2), (C) Hubei (clade 2.3.2.1a), and (D) Anhui (clades 2.3.4). Neutralization curves of antisera elicited by the Ad-H5 (prime) + rH5 (boost) regimen against (E) KAN-1 (clade 1), (F) Qinhai (clade 2.2), (G) Hubei (clade 2.3.2.1a), and (H) Anhui (2.3.4). [file Image_3.tif]

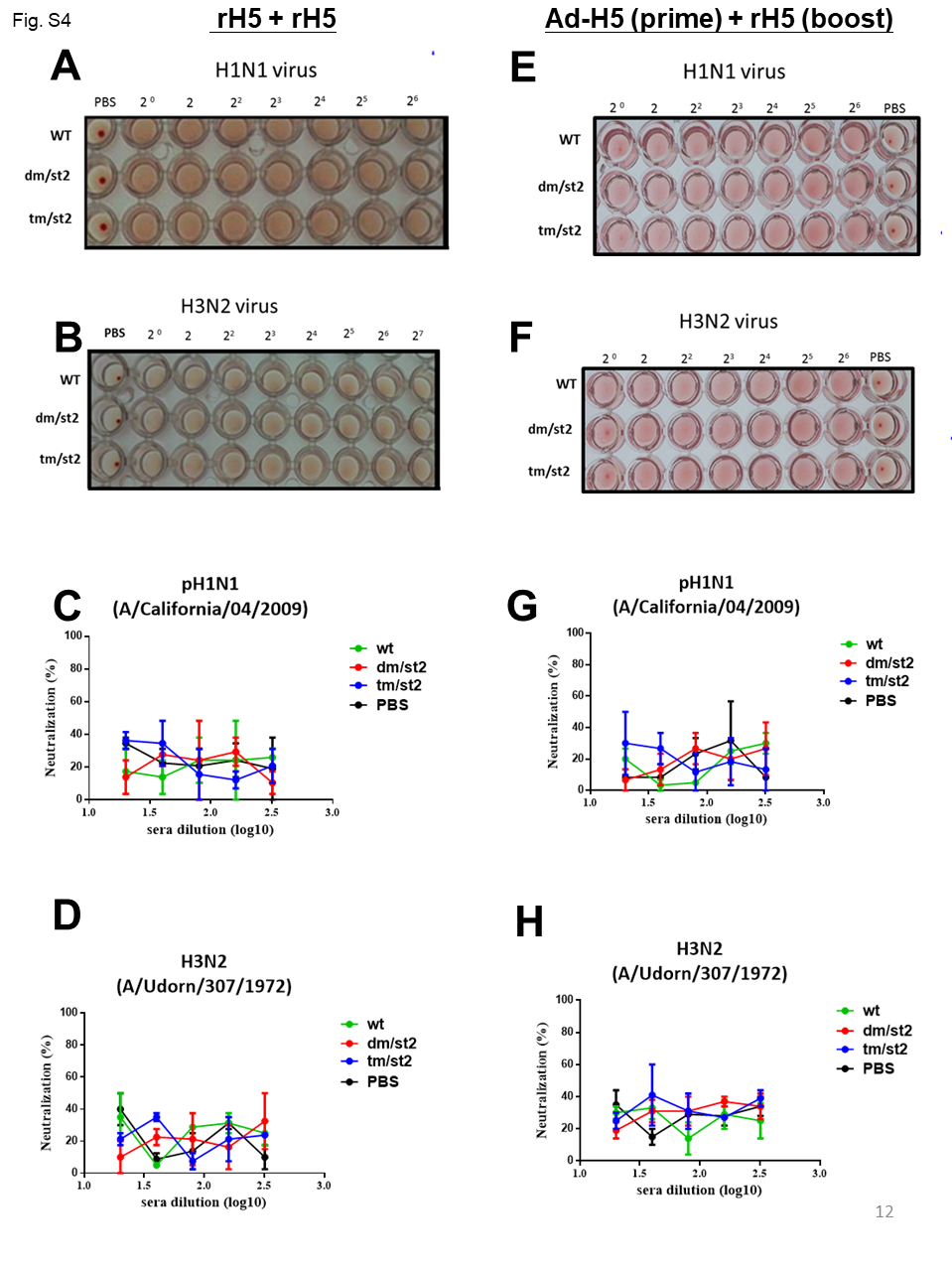

Supplement: Supplementary Figure 4 — Antisera against the heterosubtypic pH1N1 (A/California/04/2009) and H3N2 (A/Udorn/307/1972) viruses. Immunization with rH5 + rH5 regimen: (A) hemagglutinin inhibition against pH1N1, (B) hemagglutinin inhibition against H3N2, (C) PRNT neutralization curves against pH1N1, (D) PRNT neutralization curves against H3N2; Immunization with Ad-H5 (prime) + rH5 (boost) regimen: (E) hemagglutinin inhibition against pH1N1, (F) hemagglutinin inhibition against H3N2, (G) PRNT neutralization curves against pH1N1, (H) PRNT neutralization curves against H3N2. [file Image_4.tif]
